# Empagliflozin attenuates elaidic acid-exacerbated cardiac dysfunction post-myocardial infarction via suppression of the NF-κB/NLRP3 pathway

**DOI:** 10.3389/fcvm.2026.1765915

**Published:** 2026-04-22

**Authors:** Qingkai Yan, Jin Xiao, Changqing Yu, Yuehui Yin

**Affiliations:** 1 Department of Cardiology, The Second Affiliated Hospital of Chongqing Medical University, Chongqing, China; 2 Department of Cardiology, Bishan Hospital of Chongqing Medical University, Chongqing, China

**Keywords:** elaidic acid, empagliflozin, mitochondria, myocardial infarction, NF-κB/NLRP3 signaling pathway

## Abstract

**Objective:**

Myocardial infarction leads to heart failure greatly increasing the risk of adverse cardiovascular events. Empagliflozin (EMPA), a sodium-dependent glucose transporters 2 (SGLT2) inhibitor, has been demonstrated to provide effective cardiovascular protection, while the underlying metabolic mechanisms of EMPA on protecting cardiac dysfunction post-MI remain incompletely clear. The current study was conducted to investigate the metabolic effects of EMPA in hearts with cardiac dysfunction post-MI.

**Methods:**

Bioinformatics analysis revealed activation of the NF-κB/NLRP3/pyroptosis signaling pathway in AMI patients. *In vivo*, MI mice were generated and treated with empagliflozin (EMPA) or saline for 4 weeks, assessing cardiac structure and function via ultrasound and histological staining. Concurrently, Fatty acid content in cardiac tissues was evaluated from all three groups using targeted metabolomics, with an assessement of the NF-κB/NLRP3/pyroptosis signaling pathway in each group via western blot and immunohistochemistry.

**Results:**

The bioinformatics analysis was conducted using GSE97320 dataset found the activation of the NF-κB/NLRP3/pyroptosis signaling pathway in MI patients. *In vivo* experiments revealed that cardiac structural abnormalities and functional impairment in MI mice were significantly improved by EMPA treatment. Concurrently, EMPA treatment effectively suppressed activation of the NF-κB/NLRP3 signaling pathway, accompanied by reduced levels of IL-1β and IL-18, suggesting decreased levels of cardiomyocyte pyroptosis and markedly alleviated inflammatory infiltration and myocardial fibrosis. Furthermore, targeting lipid metabolism revealed marked accumulation of elaidic acid in cardiomyocytes of MI mice, which was significantly reduced in myocardial tissue following EMPA treatment. This improvement was accompanied by restoration of mitochondrial structure and function.

**Conclusion:**

EMPA treatment effectively improves cardiac structure and function in MI mice, potentially through regulating lipid metabolism and reducing tissue EA levels, thereby inhibiting NF-κB/NLRP3/pyroptosis while alleviating mitochondrial structural and functional abnormalities.

## 1 Introduction

As epidemiological studies indicated, myocardial infarction (MI) is the most common cause of heart failure. Early identification and mitigation of cardiac injury contribute to reducing the risk of adverse cardiovascular events (1–3). Previous studies have demonstrated that angiotensin-converting enzyme inhibitors, beta-blockers, and mineralocorticoid receptor antagonists can improve left ventricular remodeling after MI, including reduce left ventricular mass, and enhance systolic function. Despite significant advances in treating MI patients over the past two decades, the incidence of heart failure post-MI and adverse cardiovascular events remains high (4, 5).

In recent years, SGLT2 inhibitors have demonstrated favorable protective effects across multiple cardiovascular conditions. Santos-Gallego et al. (6) found that empagliflozin (EMPA) treatment reduced MI area by approximately 50%, while significantly improving left ventricular remodeling after 2 months with a 41%–44% increase in short-axis shortening fraction. Concurrently, empagliflozin treatment reduced wall stress and neurohumoral activity. Furthermore, Moellmann et al. (7) observed that EMPA treatment significantly improved cardiac diastolic function in db/db rats fed a high-fat diet. This effect may be associated with enhanced insulin signaling, increased cGMP-dependent phosphorylation within the myocardium, and reduced spontaneous diastolic calcium release from the sarcoplasmic reticulum (8). Clinically, trials have evaluated EMPA efficacy and safety in diabetic and non-diabetic patients with severe AMI, demonstrating that EMPA effectively improves left ventricular remodeling, reduces preload and afterload (9, 10).

Energy metabolism in cardiomyocytes is the physiological basis for maintaining the stability of the cardiac microenvironment and for cardiac systolic and diastolic function, while mitochondrial dysfunction is a major factor contributing to heart failure following myocardial infarction (11). During myocardial ischemia, changes in myocardial energy metabolism lead to reduced fatty acid oxidation and increased glucose utilization, resulting in sustained lipid accumulation in cardiomyocytes and disruption of mitochondrial metabolism, thereby exacerbating energy expenditure (12, 13). Meanwhile, current findings indicate that EMPA promotes fatty acid β-oxidation to enhance myocardial energy metabolism efficiency while simultaneously improving mitochondrial biogenesis and function, boosting mitochondrial oxidative capacity, and reducing ROS production and oxidative stress damage (14–17). Therefore, the purpose of the study was to investigate the metabolic effects of EMPA on cardiac dysfunction post-MI.

## 2 Methods

### 2.1 Collection of gene expression data from clinical patients

Peripheral blood microarray gene expression profiles from the GSE97320 dataset were retrieved from the GEO database (https://www.ncbi.nlm.nih.gov), and GPL570 was used as the series matrix file for annotation. This dataset evaluates differential expression of genes associated with AMI in northeastern China. Peripheral blood was collected from 3 AMI patients and 3 control subjects. Total RNA was extracted from each peripheral blood sample and hybridized to Affymetrix microarrays. Differential expression analysis was performed using the R package ggplot2 (version 3.3.6). Volcano plots identified genes with log2 fold change (FC) ≥ 1 or log2 FC ≤ −1, combined with an adjusted *p*-value <0.05. Functional annotation of these genes was performed using the clusterProfiler package in R. *P*-values were adjusted using the Benjamini–Hochberg (BH) method, followed by GO and KEGG enrichment analyses.

### 2.2 Animals

All animal procedures were conducted in accordance with the NIH Guide for the Care and Use of Laboratory Animals (NIH Publication, 2011 revised edition) and approved by the Animal Care and Use Committee of Chongqing Medical University. All C57BL/6J mice in this study were housed in individually ventilated cages with free access to water and food.

### 2.3 Animal groups and procedures

Eighteen mice were randomly divided into three groups: Sham, MI, and MI + EMPA, with *n* = 6 per group. Following adaptive feeding, mice in each group were anesthetized with pentobarbital (2.5%, intraperitoneal injection) and immobilized. After successful tracheal intubation, they were connected to a small animal ventilator. Electrocardiograms were recorded using ECG acquisition software. Following preparation, the left third intercostal space of the sternum was located. After thoracotomy, the anterior descending branch of the left coronary artery was ligated at the junction of its middle and distal thirds. Successful MI modeling was confirmed when the ECG recording software detected persistent ST-segment elevation. The EMPA group received 20 mg/kg/day via oral gavage, while the control group received physiological saline via oral gavage for 4 weeks. Except for the absence of coronary artery ligation, the surgical procedure in the sham group was identical to that in the MI group.

### 2.4 Echocardiography

During echocardiography, animals were anesthetized with 1.5% isoflurane and placed on a heated pad. Two-dimensional (2D) parasternal short-axis M-mode echocardiographic images were recorded. Cardiac ultrasound parameters were measured over 3–5 consecutive cardiac cycles, including left ventricular end-diastolic diameter, left ventricular end-systolic diameter, left ventricular end-diastolic volume, and left ventricular end-systolic volume. Left ventricular ejection fraction and shortening fraction were calculated to assess cardiac function.

### 2.5 ELISA analysis

The levels of the interleuckin-1β (IL-1β, EM0109), interleuckin-18 (IL-18, EM1158), tumor necrosis factor-α (TNF-α, EM0183), and interleuckin-6 (IL-6, EM0121) in cardiac tissue were detected using a kit, with all experiments conducted according to the manufacturer's instructions.

### 2.6 Histological analysis

Heart tissue isolated from animals was fixed in 4% paraformaldehyde and subsequently dehydrated. It was then embedded in paraffin. The specimen was sectioned into 3–5 μm thick slices. Hematoxylin and eosin (H&E) staining, WGA staining, and Masson's trichrome staining were conducted to evaluate morphology and infarction size in hearts post-MI.

### 2.7 TUNEL staining

The TUNEL kit (Servicebio, China) was used according to the protocol in order to evaluate the apoptosis of heart tissue. The images were captured via fluorescence microscope and analyzed using Image-Pro Plus.

### 2.8 Immunohistochemistry

In order to evaluate the expressions of Nod-like receptor protein 3 (NLRP3, 1:1000) and F4/80 (1:1000) at protein levels using the immunohistochemistry staining.

### 2.9 Targeted lipid metabolome sequencing

High-throughput targeted metabolomics was employed to detect the levels of lipid metabolites in cardiac tissues from three groups of mice.

### 2.10 Electron microscopy

Tissues and cells were first fixed with 2% paraformaldehyde and 0.2% glutaraldehyde in sodium cacodylate buffer (pH 7.4) at 37 °C for 1 h, dehydrated in a graded ethanol series and embedded. Approximately 75-nm ultrathin sections were mounted on nickel grids. The samples were then stained and visualized via a 120 kV JEOL electron microscope at 80 kV.

### 2.11 Cell culture

H9c2 cells were cultured in high-glucose Dulbecco's Modified Eagle's Medium (DMEM, Gibco), supplemented with 10% fetal bovine serum (Sigma) and 1% penicillin/streptomycin (Gibco), in an incubator at 37 °C. Meanwhile, H9c2 cells were subjected to oxygen-glucose deprivation (OGD) *in vitro* to simulate myocardial ischemia. Briefly, the complete growth medium was replaced with serum- and glucose-free DMEM, and the cells were then transferred to an incubator containing 95% N_2_ and 5% CO_2_ at 37 °C for 12 h. In control group, cells were maintained under normal conditions in standard medium. EMPA was utilized to treat H9c2 cells at 500 nM concentration (18). After OGD stimulation, H9c2 cells were treated with EA at 100 µm (19).

### 2.12 Western blotting

The primary antibodies against NLRP3 (1:1000, #AG-20B-0014-C100, Adipogen), cleaved-GSDMD (1:1000, #10137T, CST), p-NF-κB (1:1000, #82335-1-55, Proteintech), NF-κB (1:1000, #10745-1-AP, Proteintech), Cleaved Caspase-1 (1:1000, #AG-20B-0042-C100, Adipogen), GAPDH (1:1000, GB15004, Servicebio), OXPHOS (1:1000, ab110413, Abcam), Cox IV (1:1000, 11242-1-ap, Abcam) were used for western blot analysis. All protein levels were normalized to the levels of GAPDH via Image J.

### 2.13 Oxidative stress analysis

*In vitro*, the levels of ROS were measured by dichlorodihydrofluorescein diacetate (DCFH-DA) staining. The cells were incubated with DCFH-DA at a concentration of 5 μmol/L at 37 °C for 30 min. After that, the images were captured with a fluorescence microscope.

### 2.14 Statistical analysis

The data are presented as the mean ± standard error (SEM). This refers to biological replicates, not technical replicates. The normality of the distribution of the data for the analysis of *in vivo* experiments was evaluated via the Shapiro–Wilk test. A P value of greater than 0.05 indicated that the data were approximately normally distributed for each group. To identify meaningful differences between groups, two-tailed Student's *t*-tests were used to compare two groups, and one-way ANOVA followed by Tukey's *post hoc* test was used to compare more than two groups. Statistically significant differences were obtained at *p* < 0.05. All data analyses were performed using GraphPad Prism 9.0 software (San Diego, CA, USA).

## 3 Results

### 3.1 Activation of the NF-κB/NLRP3 signaling pathway in patients with MI

**Figure 1:**
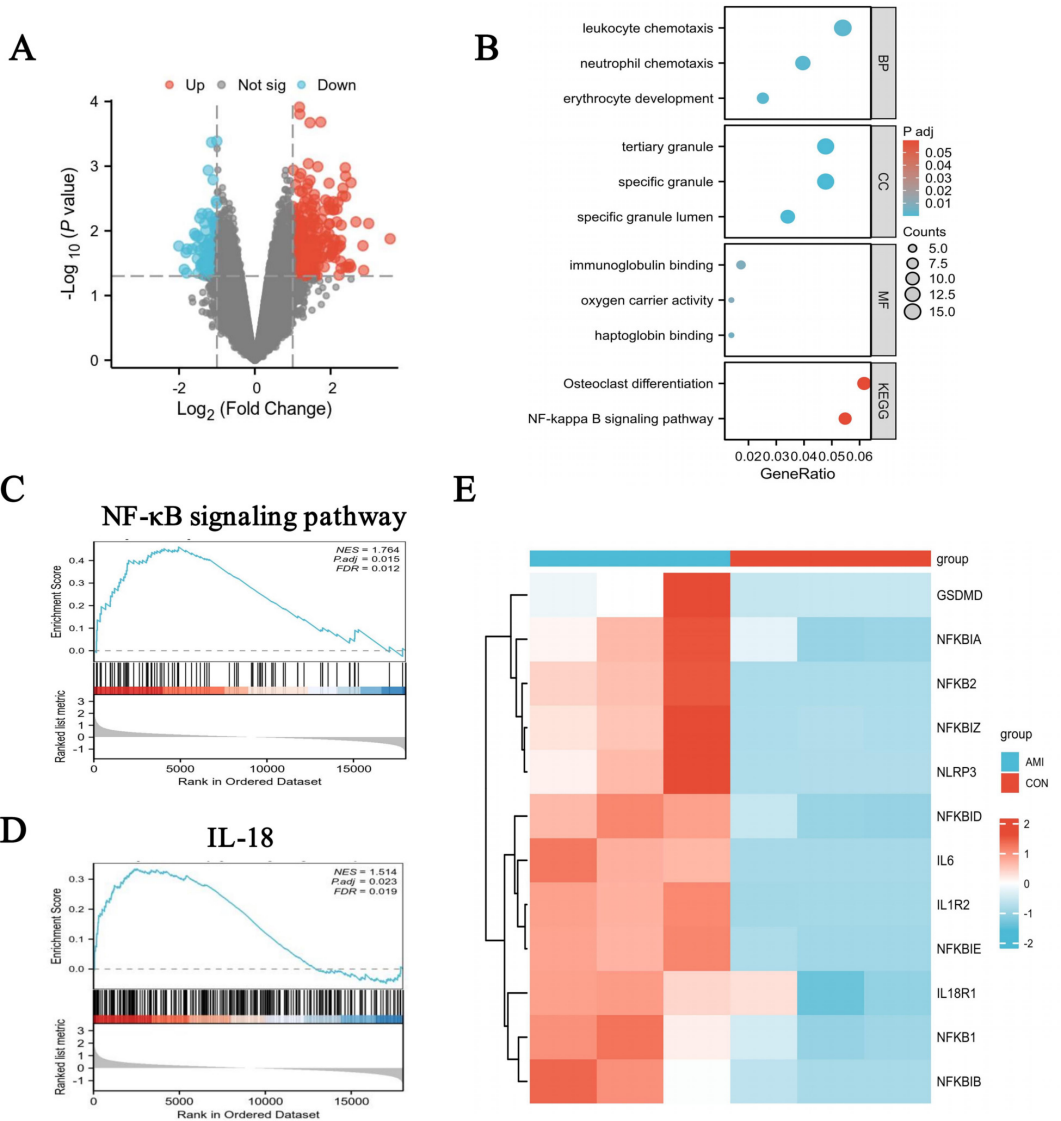
Activation of the NF-κB/NLRP3 signaling pathway in patients with MI based on bioinformatics analysis using the GSE97320 dataset enrolling serum from patients of MI and healthy. **(A)**, Volcano plot. **(B)**, KEGG enrichment. **(C)**, GSEA analysis of NF-κB signaling pathway. **(D)**, GSEA analysis of IL-18 signaling pathway. **(E)** Heat map of gene expressions in upstream and downstream of NF-κB/NLRP3 signaling pathway (AMI patients, *n* = 3; Healthy people, *n* = 3).

The bioinformatics analysis using the GSE97320 dataset was conducted, revealing 334 differentially expressed genes in the MI cohorts compared to healthy controls (Figure 1A). Further GO enrichment analysis showed significant enrichment of these genes in pathways related to inflammatory activation, immune response, and oxidative stress. The KEGG enrichment analysis indicated marked enrichment in the NF-κB signaling pathway (Figure 1B). Concurrently, GSEA enrichment analysis revealed significant positive enrichment of the NF-κB signaling pathway in MI patients (NES = 1.764, FDR = 0.012, *p*.adj = 0.015) (Figure 1C). and the IL-18 signaling pathway was also significantly positively enriched in AMI patients (NES = 1.514, FDR = 0.012, *p*.adj = 0.023) (Figure 1D). Subsequently, a heatmap analysis of NF-κB pathway gene expression patterns was conducted, including analysis of inflammatory factors, *Nfkb*, and *Gsdmd* (Figure 1E). Results revealed widespread upregulation of *Nfkb*, *Nlrp3*, Il6, *Il18*, and *Gsdmd* expressions in MI patients, indicating activation of the NF-κB/NLRP3/pyroptosis signaling pathway in these patients.

### 3.2 EMPA therapy improves structural and functional damage in the hearts of MI mice

**Figure 2:**
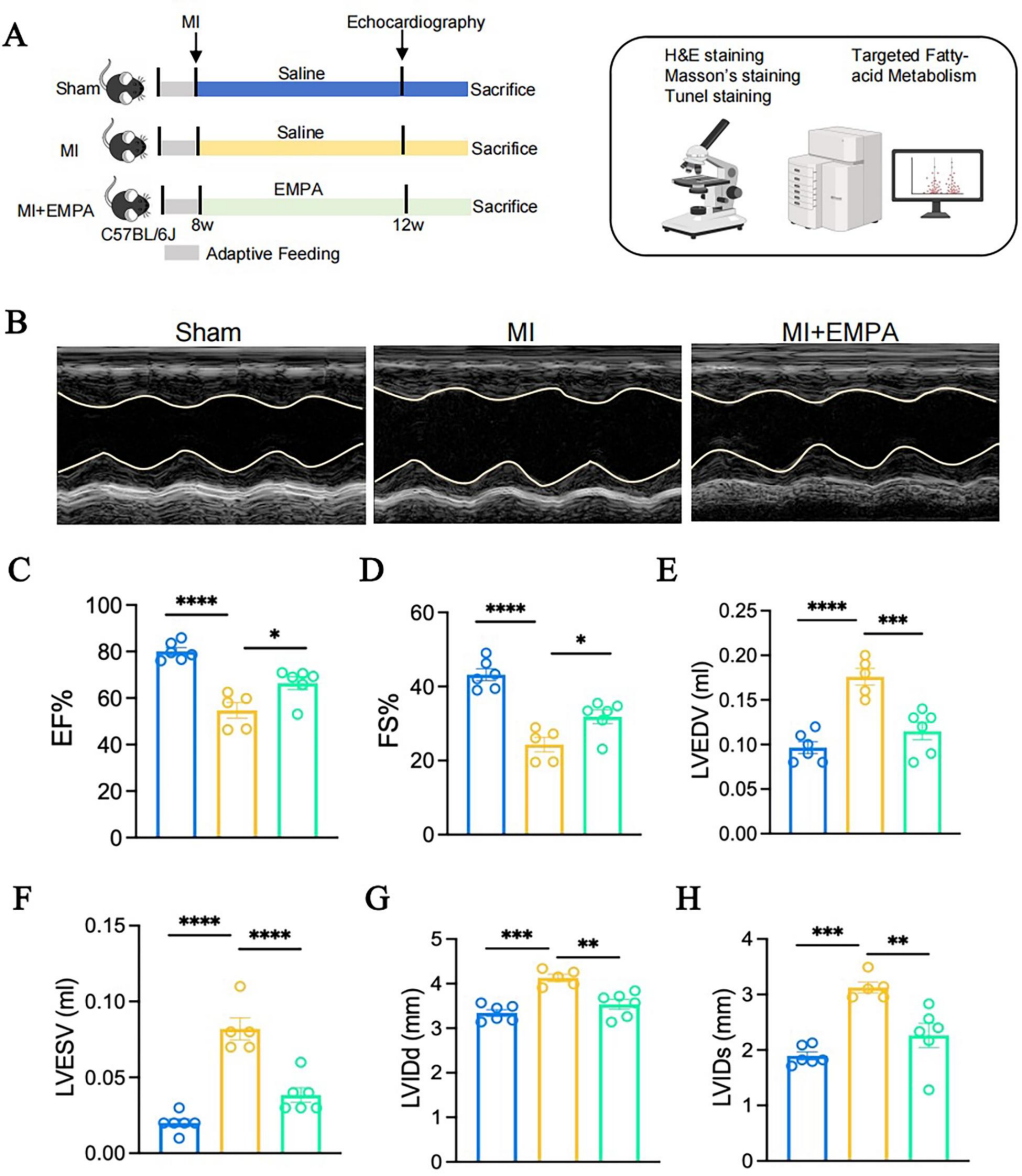
EMPA inhibits dysfunction of cardiac structure and function-post myocardial infarction. **(A)** The experimental setup. **(B)** Representative echocardiography images from 3 groups. **(C)** Ejection fraction of the mice in 3 groups. **(D)** Fractional shortening of the mice in 3 groups. **(E–H)** The structure of the mice in 3 groups, including LVEDV **(E)**, LVESV **(F)**, LVIDd **(G)**, and LVIDs **(H)** (*n* = 6 per group; *p < 0.05, **p < 0.01, ***p < 0.001, ****p < 0.0001).

Since NF-κB/NLRP3 is a key factor contributing to post-MI cardiac dysfunction, effectively inhibiting NF-κB/NLRP3 may be crucial for improving cardiac dysfunction following MI. In recent years, empagliflozin has been found to possess cardioprotective effects independent of its hypoglycemic action, with improvement in substance and energy metabolism. To further investigate its impact on metabolism and the underlying mechanisms in ischemic heart disease, we treated mice with either EMPA or saline and performed cardiac echocardiography on all groups after 4 weeks (Figures 2A,B). Results revealed significantly reduced EF and FS values in MI mice after 4 weeks, while EMPA treatment markedly improved cardiac function in MI mice (*p*-value <0.05). Concurrently, cardiac structural observations demonstrated that 4 weeks of EMPA treatment significantly improved cardiac structure in MI mice (Figures 2C,H).

### 3.3 EMPA inhibits NF-κB/NLRP3/pyroptosis signaling pathway to alleviate myocardial fibrosis in MI mice

**Figure 3:**
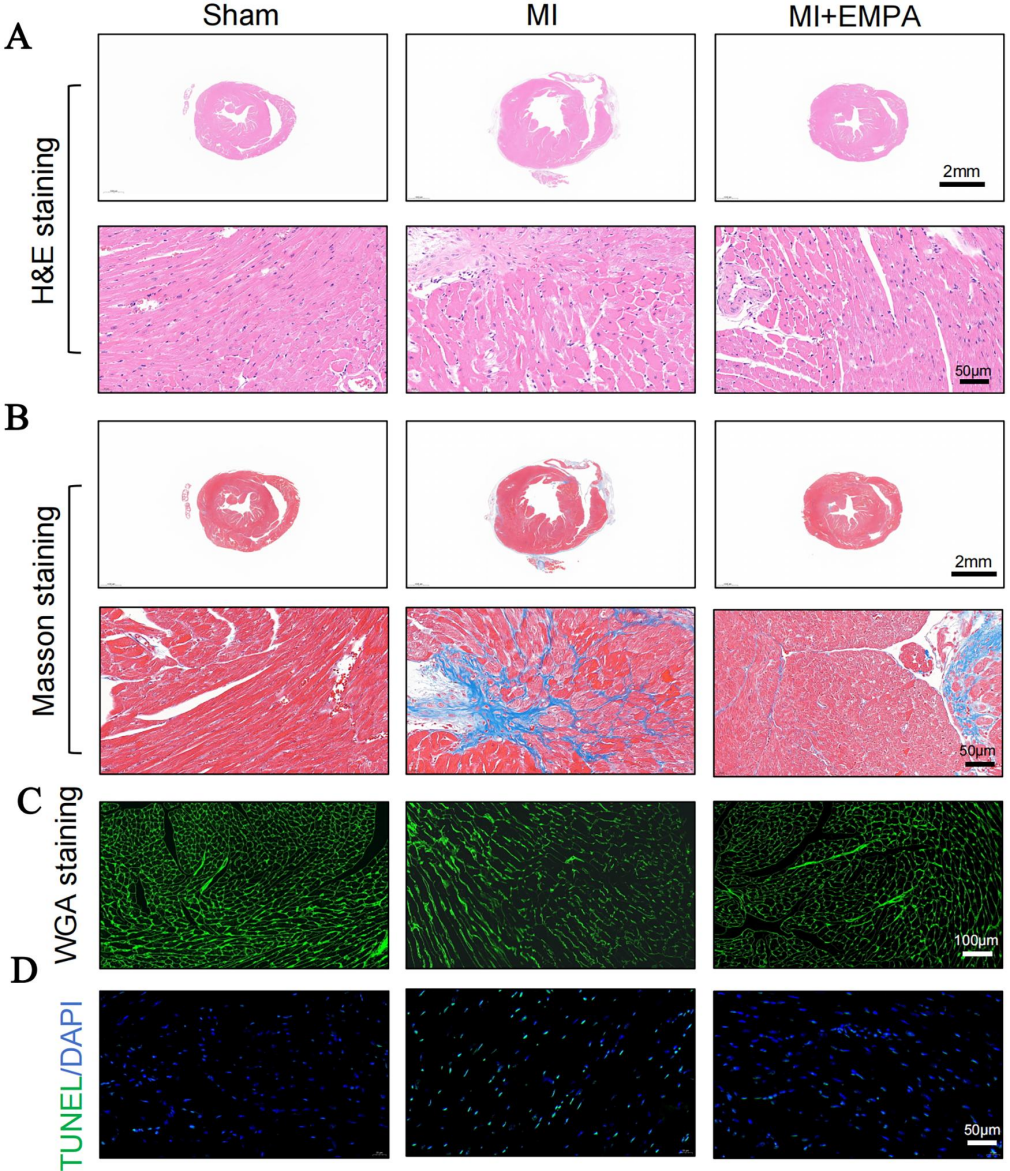
EMPA ameliorate cardiac morphology and cardiac fibrosis. **(A)** Representative images of HE staining for heart tissue. **(B)** Representative images of Masson's staining for heart tissue. **(C)** Representative images of WGA staining for heart tissue. **(D)** Representative images of TUNEL staining for heart tissue.

Meanwhile, the histological staining also revealed marked inflammatory cell infiltration in the MI group mice. Masson staining demonstrated significantly increased myocardial fibrosis. EMPA treatment markedly reduced inflammatory infiltration and fibrosis levels in cardiomyocytes (Figures 3A,C). Further TUNEL staining results supported that EMPA treatment significantly improved cardiomyocyte apoptosis levels in the MI group mice (Figure 3D).

**Figure 4:**
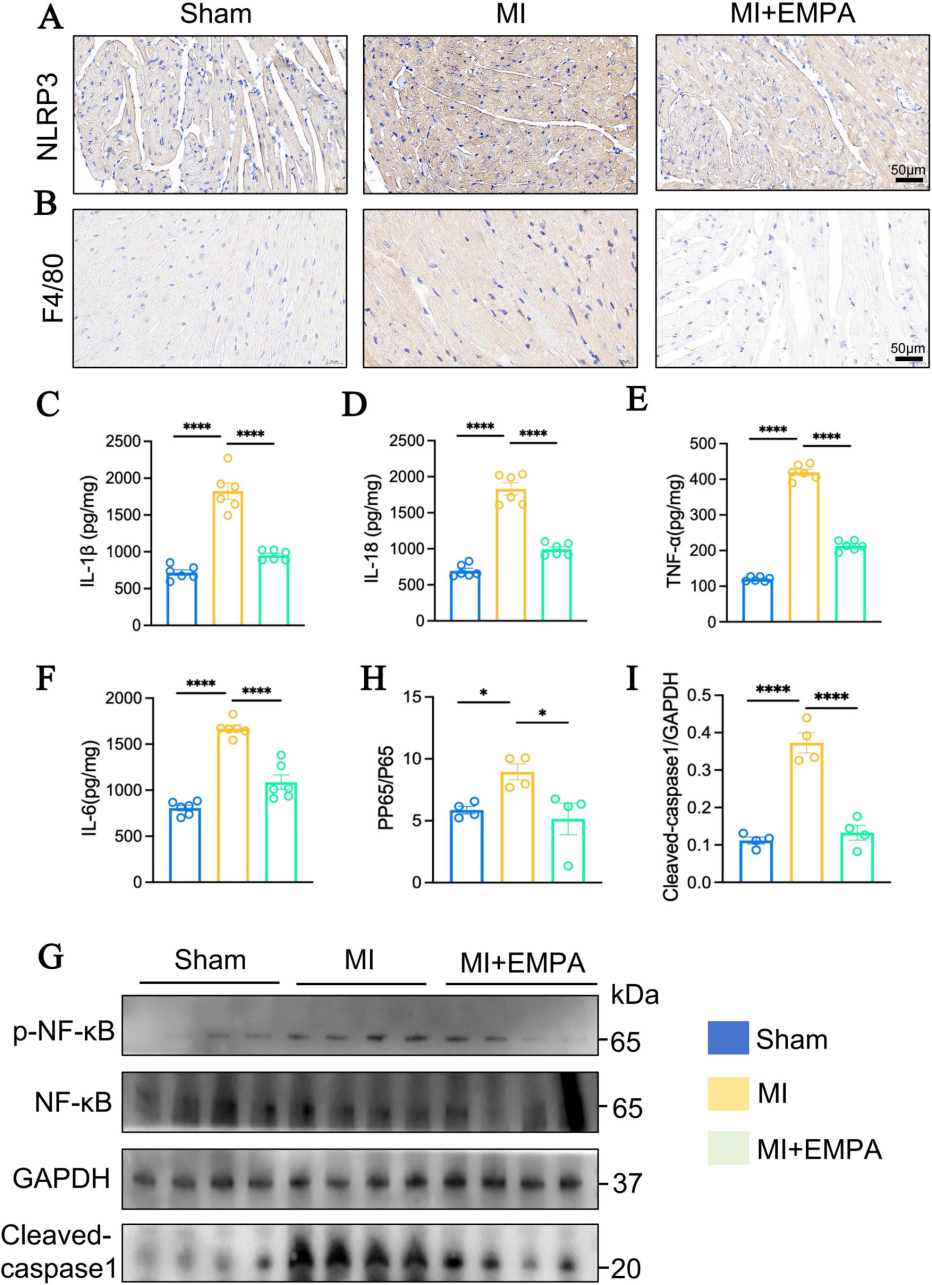
EMPA treatment inhibited NF-κB/NLRP3/pyroptosis signaling pathway in hearts after MI. **(A)** IHC demonstrating that NLRP3 expressions at protein level in hearts from each group. **(B)** IHC demonstrating that F4/80 in hearts from each group. **(C)** The concentrations of IL-1β in hearts from 3 groups by ELISA analysis. **(D)** The concentrations of IL-18 in hearts from 3 groups by ELISA analysis. **(E)** The concentrations of TNF-α in hearts from 3 groups by ELISA analysis. **(F)** The concentrations of IL-6 in hearts from 3 groups by ELISA analysis (*n* = 6 per group; *p < 0.05, **p < 0.01, ***p < 0.001, ****p < 0.0001). **(G)** The relative protein levels of P-NF-κB, NF-κB, Cleaved-caspase 1 and GAPDH in the heart tissue were detected using western blot assay. **(H,I)** Densitometric quantification is shown for P-NF-κB/NF-κB and Cleaved-caspase 1 (*n* = 4 per group; *p < 0.05, **p < 0.01, ***p < 0.001, ****p < 0.0001).

To investigate the NF-κB/NLRP3 signaling pathway, immunohistochemical analysis revealed that EMPA treatment significantly reduced NLRP3 protein expression in MI group cardiomyocytes (Figure 4A), accompanied by a reduction of immunocyte infiltration which was evaluated by F4/80 (Figure 4B) and reduced levels of inflammatory factors (Figures 4C,F). Meanwhile, western blotting also proved the elevated NF-κB phosphorylation and up-regulation of Cleaved-caspase1 protein expressions in hearts after MI, which could be abolished by EMPA treatment (Figures 4G,I). These findings suggest that EMPA treatment suppresses the NF-κB/NLRP3/pyroptosis signaling pathway in MI group mouse cardiomyocytes, thereby alleviating myocardial fibrosis.

### 3.4 EMPA exerted the effects through alleviating fatty acid metabolism disorders and reducing elaidic acid accumulation

**Figure 5:**
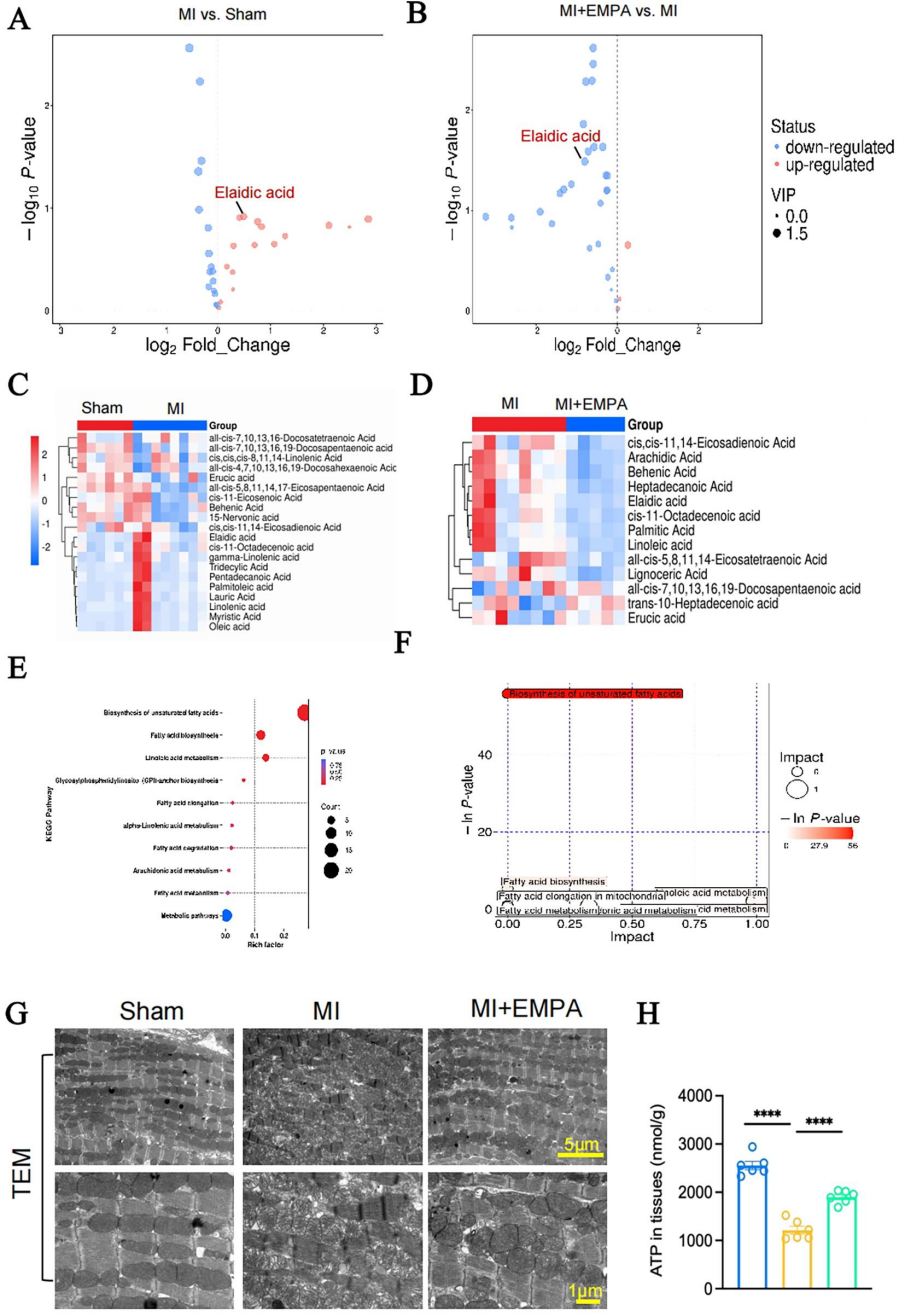
EMPA reduced the accumulation of elaidic acid in cardiac tissues from MI mice. **(A)** Volcano plot of differently fatty acids in hearts from MI and sham groups. **(B)** Volcano plot of differently fatty acids in hearts from MI mice with or without EMPA treatment. **(C)** Heat map of the top 20 fatty acids in hearts from MI and sham groups. **(D)** Heat map of the top 20 fatty acids in hearts from MI mice with or without EMPA treatment. **(E,F)** KEGG enrichment. **(G)** Transmission electron microscopy (TEM) observation of myocardial cell and mitochondrial structures in each group. **(H)** The ATP contents in cardiac tissues from each group (*n* = 6 per group; ****p < 0.0001).

Under normal conditions, over 90% of the ATP required for myocardial cell metabolism is generated through aerobic oxidation, primarily via fatty acid oxidation (FAO). Multiple studies now support the role of fatty acid metabolic dysfunction and its associated metabolites in causing cardiovascular damage. Therefore, we employed targeted fatty acid metabolomics to analyze cardiac tissues from three mouse groups. Results revealed alterations in multiple fatty acid levels in the MI group. Interestingly, following EMPA treatment, most fatty acids decreased in MI myocardial tissue, including arachidic acid, behenic acid, heptadecanoic acid, and elaidic acid (EA) (Figures 5A,D). Among these fatty acids, the change in elaidic acid was most pronounced in the MI group. Further enrichment analysis revealed significant enrichment in unsaturated fatty acid synthesis (Figures 5E,F).

Since EA is a trans oleic acid, it may be associated with mitochondrial damage in cardiomyocytes. Therefore, we examined the structure and function of myocardial cell mitochondria. In the MI group, we observed disorganized mitochondrial arrangement in cardiac tissue, accompanied by mitochondrial cristae disruption and vacuolation, z-line disruption in cardiomyocytes, and a significant decrease in ATP content. Following EMPA treatment, mitochondrial structural disorganization and arrangement were improved, while ATP content significantly increased (Figures 5G,H).

### 3.5 The enhanced elaidic acid accumulation aggravated mitochondrial dysfunction after myocardial ischemia via NF-κB/NLRP3/pyroptosis signaling pathway

**Figure 6:**
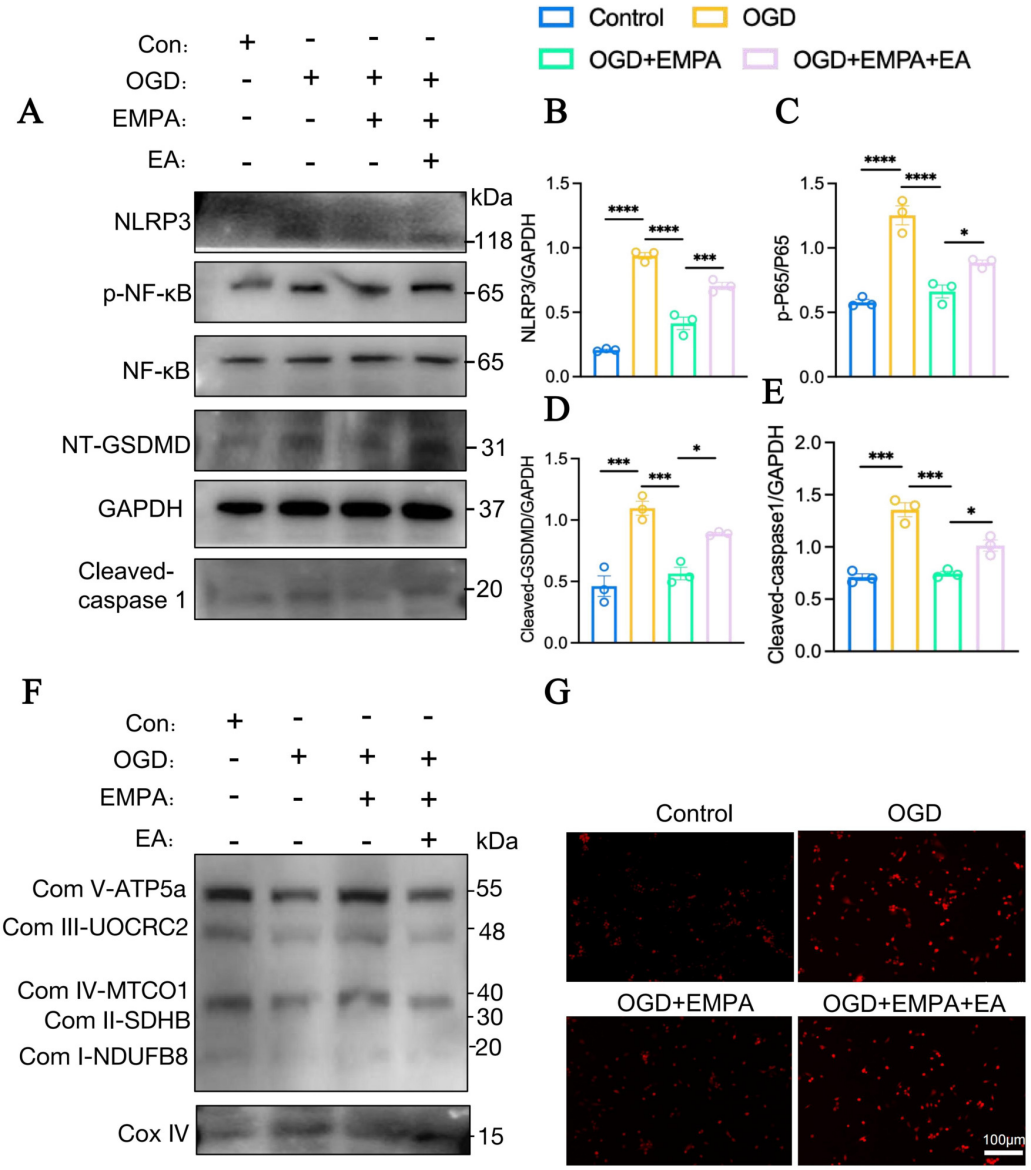
The accumulation of EA aggravated mitochondrial dysfunction and activation of NF-κB/NLRP3/pyroptosis signaling pathway. **(A)** The relative protein levels of NLRP3, NF-κB, p-NF-κB, Cleaved-GSDMD, Cleaved-caspase1 and GAPDH in H9c2 cell were detected using western blot assay. **(B)** The densitometric quantification of NLRP3. **(C)** The densitometric quantification of p-NF-κB/NF-κB. **(D)** The densitometric quantification of Cleaved-GSDMD. **(E)** The densitometric quantification of Cleaved-caspase1. **(F)** The relative protein levels of ETC complexes using western blot assay. **(G)** DCFH-DA detected the ROS levels (*n* = 3, *p < 0.05, **p < 0.01, ***p < 0.001, ****p < 0.0001).

To further clarify the mechanism of EA in myocardial ischemia, we identified OGD for 12 h to simulate myocardial ischemia *in vitro*. As immunoblotting shown that NLRP3, p-NF-κB, Cleaved-GSDMD, and Cleaved-caspase 1 in cells stimulated with OGD. Furthermore, EMPA treatment greatly decrease the expressions of NLRP3, p-NF-κB, Cleaved-GSDMD, and Cleaved-caspase 1 at protein levels. Moreover, for the cells under OGD stimulation and treated with EMPA, it discovered markedly over-expressed protein levels of NLRP3/NF-κB pathway (Figures 6A,E).

Herein, in order to explore the role of EA stimulation in OGD-induced myocardial injury and mitochondrial funciton, we further confirmed that the protein expressions of mitochondrial electron transport chain (ETC) complexes. It revealed the ETC complexes decreased prominently, which were elevated by EMPA incubation. Interestingly, the alleviation by EMPA treatment could be abolished after EA stimulation in H9c2 (Figure 6F). Meanwhile, the ROS levels also approved the effects of EA stimulation for aggravating mitochondrial dysfunction in myocardial ischemia (Figure 6G).

## 4 Discussion

Heart failure (HF) represents the terminal stage of various cardiac diseases, significantly impacting patients’ quality of life and survival rates. Ischemic heart disease and hypertension are the primary causes of HF (20). Treatment for heart failure resulting from ischemic heart disease requires a combination of revascularization and pharmacotherapy. Since cardiac remodeling and the onset and progression of HF are believed to be associated with impaired substrate utilization and energy deficiency in cardiomyocytes (21), improving material and energy metabolism in the failing heart is crucial for managing heart failure caused by ischemic heart disease.

Using bioinformatics analysis, we identified the rapid activation of the NF-κB/NLRP3 signaling pathway in patients suffered from MI. As studies have demonstrated, the NF-κB/NLRP3 signaling pathway plays a critical role in mice with heart failure following MI, and that inhibiting this pathway effectively reduces inflammatory infiltration and damage in cardiomyocytes (22, 23).

In recent years, SGLT2 inhibitors, particularly EMPA, have garnered significant attention for their pronounced cardioprotective effects. Both the EMPA-REG OUTCOME (24) and EMPEROR-Reduced/Preserved trials (25) confirmed that EMPA reduces cardiovascular mortality and the risk of hospitalization for heart failure, with these benefits independent of its glucose-lowering effects. Concurrently, it has suggested that EMPA may inhibit NLRP3 inflammasome activation and enhance energy metabolism by improving mitochondrial function (26). However, the mechanism by which EMPA improves myocardial cell mitochondrial function during myocardial infarction through the NF-κB/NLRP3/pyroptosis pathway remains unclear.

Firstly, it employed bioinformatics analysis to demonstrate that the NF-κB/NLRP3/pyroptosis pathway mediating inflammatory responses is significantly activated in MI patients. The NLRP3 inflammasome serves as a key regulator of pyroptosis (27, 28). Its excessive activation is closely associated with pathological processes in various diseases such as diabetes, atherosclerosis, heart failure, and chronic kidney disease (29, 30). Indeed, the Toll-like receptor (TLR)/NF-κB signaling pathway promotes NLRP3 activation, as well as the transcription of pro-caspase-1 and pro-IL-1β. Furthermore, NLRP3 assembles with caspase-recruitment domain-containing and caspase-1-precursor-joining proteins, mediating the secretion of proinflammatory cytokines including IL-1β and IL-18 (31).

Currently, cardiac structural abnormalities and functional impairment in the MI group were found significantly improved by EMPA treatment. Concurrently, EMPA treatment effectively suppressed activation of the NF-κB/NLRP3 signaling pathway, accompanied by reduced levels of IL-1β and IL-18, suggesting decreased levels of cardiomyocyte pyroptosis and significantly alleviated inflammatory infiltration and myocardial fibrosis. Furthermore, targeting lipid metabolism revealed marked accumulation of elaidic acid in cardiomyocytes of MI mice, which significantly decreased in myocardial tissue following EMPA treatment. This was accompanied by improved mitochondrial structure and function. EA is a trans fatty acid, the levles of which is correlate with cardiovascular diseases such as diabetes, coronary heart disease, and obesity. Research also highlights that serum EA is a key factor in insulin resistance and diabetes progression (32). Interestingly, a multicenter prospective cohort study revealed a significant association between serum NEFA concentrations and coronary heart disease mortality as well as sudden non-fatal myocardial infarction. Among these, elaidic acid showed a positive correlation with MI events (HR = 1.46, 95% CI = 1.01–2.12, *p* = 0.0445) (33). In earlier work, Mitmesser et al. incubated cells with different fatty acids and observed effects on lipoprotein secretion and composition. Results showed that HepG2 cells incubated with elaidic acid secreted higher levels of ApoB protein, and the secreted lipoprotein particles were significantly smaller. This suggests EA may be associated with the onset and progression of CHD (19, 34). The fatty acids are currently recognized to activate various inflammatory signaling pathways, thereby increasing cardiovascular disease risk. EA has been demonstrated to promote apoptosis by inducing ROS accumulation, endoplasmic reticulum stress, and inflammatory activation (35). Concurrently, Lu et al. demonstrated that EA induces hepatocyte pyroptosis via NLRP3 inflammasome activation (36), suggesting that reducing EA levels may effectively mitigate pyroptosis and inflammatory infiltration. This study revealed significantly elevated EA levels in myocardial tissues of MI mice, accompanied by activation of the NF-κB/NLRP3/pyroptosis signaling pathway, marked mitochondrial structural and functional impairment, and pronounced inflammatory cell infiltration and myocardial fibrosis. EMPA treatment significantly reduced Elaidic acid levels in MI mouse cardiac tissue, inhibited NF-κB/NLRP3/pyroptosis, improved mitochondrial structure and function, and restored impaired cardiac structure and function. Previous studies have suggested EA may cause mitochondrial damage by altering the electrophysiological properties of the voltage-dependent anion channel (VDAC) (37).

Therefore, *in vivo* and *in vitro*, EMPA reduced EA levels in MI mouse hearts, with suppressed the NF-κB/NLRP3/pyroptosis signaling pathway. It contributed to the improvement in mitochondrial structure and function, further restoring cardiac structure and function. While some limitations were still existed: (1) A further study should be conducted to identify the dynamic changes of EA during the MI process; (2) The early monitoring of EA levels should be monitored, and the effects of EA intake should be further investigated in MI patients.

## 5 Conclusions

In conclusion, the current study reported that EMPA treatment effectively improves cardiac structure and function in MI mice, potentially through mechanisms involving lipid metabolism regulation, reduced EA levels in tissues, inhibition of NF-κB/NLRP3/pyroptosis, and concurrent mitigation of mitochondrial structural and functional abnormalities.

## Data Availability

The datasets presented in this study can be found in online repositories. The names of the repository/repositories and accession number(s) can be found in the article/Supplementary Material.
